# Analysis of the correlation between non-alcoholic fatty liver disease and the risk of colorectal neoplasms

**DOI:** 10.3389/fphar.2022.1068432

**Published:** 2022-11-09

**Authors:** Shujun Ye, Yang Liu, Te Zhang, Huijin Feng, Yanqing Liu, Lianjun Ma

**Affiliations:** ^1^ Endoscopy Center, China-Japan Union Hospital of Jilin University, Changchun, Jilin, China; ^2^ Anesthesiology Department, China-Japan Union Hospital of Jilin University, Changchun, Jilin, China; ^3^ Herbert Irving Comprehensive Cancer Center, Columbia University, New York, NY, United States

**Keywords:** NAFLD, CRN, CRA, CRC, ferroptosis, cuproptosis

## Abstract

This study aims at assessing the potential association between non-alcoholic fatty liver disease (NAFLD) and colorectal neoplasms (CRN). PubMed, Cochrane Library, and Embase were searched for cohort studies. 14 cohort studies with a total population of 38,761,773 were included for meta-analysis after selection. The results showed that NAFLD is related to an increased risk of CRN (OR = 1.23; 95% CI: 1.14–1.32; I^2^ = 70.7%, *p* < 0.001). In the subgroup analysis, NAFLD were found to be the independent risk factor of colorectal adenoma (CRA) (OR = 1.29; 95% CI = 1.15–1.45; I^2^ = 66.4%) and colorectal cancer (CRC) (OR = 1.13; 95% CI = 1.12–1.15; I^2^ = 69.4%). There is no close correlation between smoking status of NAFLD patients and CRN. Interestingly, bioinformatics analysis revealed that there were overlap of dysregulated gene sets among NAFLD, CRC, and two recently identified regulated cell death types, ferroptosis and cuproptosis, respectively. Our meta- and bioinformatics analysis shows that NAFLD increases the risk of CRN. Ferroptosis and cuproptosis may be the critical links between NAFLD and CRN, respectively. These findings here support that NAFLD is necessary to be considered as an emerging risk factor for CRN.

## Introduction

Colorectal neoplasms (CRN) include colorectal adenoma (CRA) and colorectal cancer (CRC), with CRA transformation typically leading to CRC. CRC has been estimated the third common cancer type, and the second most common cause of cancer-associated mortality worldwide (Sung et al., 2021). In the past few decades, the incident of CRC has increased in most countries and is still on the rise (Sung et al., 2021). The development of screening techniques boosts the diagnosis of CRC (Ladabaum et al., 2020). However, the increase of CRC incidence (especially among younger persons) around the world indicates that severe challenges remain to control CRC. Many risk factors such as genetic factor, dietary habit, and the living environment are known to contribute to the occurrence of CRC (Sclafani et al., 2013; Budhathoki et al., 2015). Exploration of more risk factors underlying CRN (particularly CRC) initiation and development is a necessary and efficient way to benefit the prevention, diagnosis, and treatment of CRN.

As the economy develops, people have changed a lot in their living habits and diet structures, which partly lead to the higher and higher incidence of non-alcoholic fatty liver disease (NAFLD). NAFLD is among the most common chronic liver diseases worldwide, with an estimated prevalence of 25.2%, making it a severe public problem that endangers human health (Younossi et al., 2016). NAFLD is closely associated with insulin resistance, metabolic syndrome (MetS), diabetes, and cardiovascular disease, suggesting that NAFLD is a multi-system disease with extrahepatic complications (Armstrong et al., 2014; Byrne and Targher, 2015; Chacko and Reinus, 2016). Importantly, several independent risk factors for NAFLD, such as hyperlipidemia, obesity, and diabetes, have also been identified as potential risk factors for CRN (Assy et al., 2000; Donati et al., 2004). Hence, we speculate that NAFLD may be related to an increased risk of CRN.

Ferroptosis and cuproptosis are two novel regulated cell death modalities identified recent years. Ferroptosis is caused by the imbalance of diverse cellular metabolic processes, while cuproptosis results from copper-induced proteotoxic stress (Stockwell, 2022; Tsvetkov et al., 2022). Since its discovery, ferroptosis has become one of the most remarkable research topic. It has been demonstrated to involve in a variety of disorders, including NAFLD and CRC. For cuproptosis, this field is just in its infancy. Do these two cell death modes have any physiological or pathological relevance to NAFLD-related CRN? The answer to this question will not only deepen our understanding of NAFLD and CRN, but also provide novel therapeutics for both NAFLD and CRN.

Here we performed systematic analysis of the existing population-based studies to check the association between NAFLD and CRN. We revealed that NAFLD is a novel independent factor for CRN (particularly CRC). In addition, we identified that ferroptosis and cuproptosis may link NAFLD with CRC mechanistically and therapeutically, respectively.

## Materials and methods

The meta-analysis section fulfills the standard in the Preferred Reporting Items for Systematic Reviews and Meta-Analyses (PRISMA) (Page et al., 2021). The approval number of the protocol from the International Prospective Register of Systematic Reviews (PROSPERO) platform is CRD42022335269.

### Literature searching

We searched the PubMed, Embase, and Cochrane Library databases by using Medical Subject Headings (MeSH) terms and keywords as follows: (“Non-alcoholic Fatty Liver Disease” OR “Non alcoholic Fatty Liver Disease*” OR “NAFLD” OR “Fatty Liver Nonalcoholic*” OR “Liver Nonalcoholic Fatty*” OR “Nonalcoholic Fatty Liver*” OR “Nonalcoholic Steatohepatiti*”) AND (“Colorectal Neoplasms” OR “Colorectal Neoplasm*” OR “Colorectal Tumor*” OR “Colorectal Cancer*” OR “Colorectal Carcinoma*”). The detailed search strategy is described in Supplementary Tables S1, S2.

### Eligibility criteria

Following criteria were used to incorporate studies: 1) cohort study; 2) population-based study; 3) patients in the exposed group are with NAFLD, but in the control group are not; and 4) the outcome is the risk of CRN (with an adjusted odds ratio (OR)).

### Exclusion criteria

Exclusion criteria were as follows: 1) conference abstracts, meta-analysis, reviews, and study protocols; 2) duplicate publications; 3) not cohort studies; and 4) studies without OR estimate with corresponding 95% confidence interval (CI).

### Data extraction

Data were extracted by using predesigned forms according to the guideline for data extraction for systematic reviews and meta-analysis (Taylor et al., 2021).

### Risk of bias assessment

The Newcastle-Ottawa scale (NOS) was used to assess the quality of cohort studies (Stang, 2010). Star ratings range from 0 to 9 were used to evaluate the quality of cohort studies: star numbers for selection of patients and measurement of exposure, comparability, and assessment of outcomes and follow-up are 4, 2, and 3, respectively. The more stars obtained, the higher the quality of literature is. Star ratings of 0–3, 4–6, 7–9 indicated the low, moderate, and high literature quality, respectively.

### Statistical analysis

OR and 95% CI were used to measure the relationship between NAFLD and the risk of CRN. The chi-square test and I^2^ value were used to evaluate the data heterogeneity from different studies. Stata statistical software version 16.0 was used throughout the meta-analysis.

### Bioinformatics data acquisition

We downloaded the GSE89632 dataset for NAFLD and TCGA-COAD dataset for CRC from NCBI and TCGA, respectively. 564 ferroptosis-associated genes were got from the FerrDb. According to the previously study (Tsvetkov et al., 2022), 347 potential cuproptosis-related genes were identified and downloaded. Limma package was introduced for differentially expressed gene (DEG) analysis. In GSE89632 dataset, log2 (fold change) > 0.5 or log2 (fold change) < −0.5, *p* < 0.05, were considered statistically significant, while in TCGA-COAD dataset, log2 (fold change) > 1 or log2 (fold change) < −1, *p* < 0.05 was considered significant difference. R software was used to draw separate volcano plots. Venn diagram package was used for Venn diagram analysis.

## Results

### Search literature results

**FIGURE 1:**
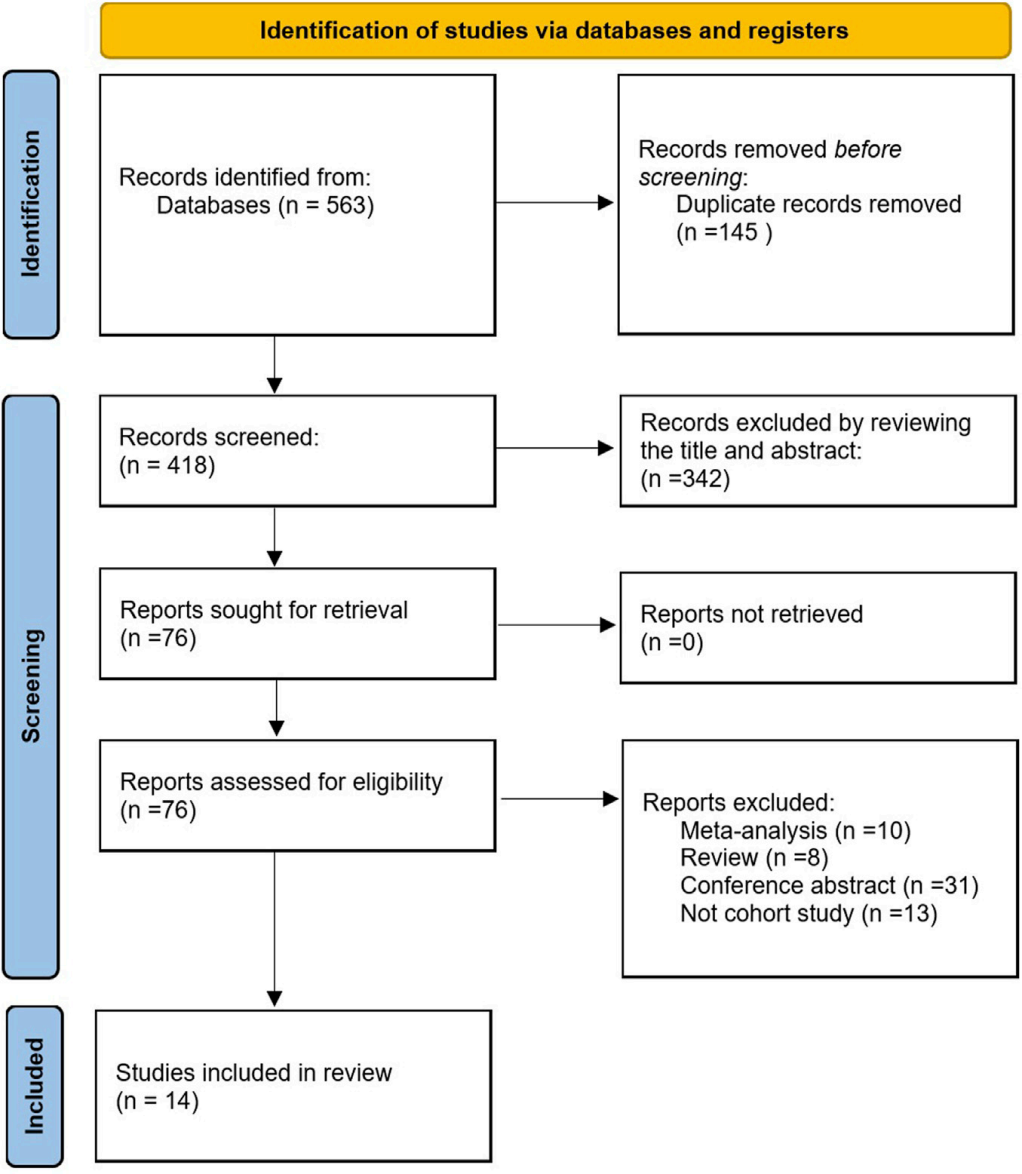
Literature screening flowchart.

According to the selection strategy, we obtained 563 literatures, among which 145 duplicate literatures were excluded. In addition, according to their titles and abstracts, 342 irrelevant articles were excluded, and 76 related articles were concluded to be thoroughly analyzed. After excluding reviews, meta-analysis, conference abstracts, and those that were not cohort studies, 14 papers (Stadlmayr et al., 2011; Wong et al., 2011; Lee et al., 2012; Lin et al., 2014; Bhatt et al., 2015; Kim et al., 2018; Ze et al., 2018; Cho et al., 2019; Hamaguchi et al., 2019; Choi et al., 2020; Lee et al., 2020; Kim et al., 2021; Wang et al., 2021; Lee et al., 2022) were included in this meta-analysis. This process is presented in Figure 1.

### Characteristics and quality of the selected studies

This batch of 14 cohort studies cover a total population of 38,761,773, which were published from 2011 to 2022. Their sample sizes range from 380 to 21,592,374 individuals, the age of participants range from 18 to 84, and the follow-up period ranged from 3 to 10.1 years. Eight studies were conducted in Korea (Lee et al., 2012; Kim et al., 2018; Ze et al., 2018; Cho et al., 2019; Choi et al., 2020; Lee et al., 2020; Kim et al., 2021; Lee et al., 2022), three in China (Wong et al., 2011; Lin et al., 2014; Wang et al., 2021), one in the United States (Bhatt et al., 2015), one in Australia (Stadlmayr et al., 2011), and one in Japan (Hamaguchi et al., 2019). Major characteristics of these studies are listed in Table 1.

**TABLE 1:** Basic characteristics of the included studies.

Author	Year	Country	Gender	Age (years)	Simple size	Study period	Confounders adjust
Choi et al. (2020)	2020	Korea	Male: 10,489,028 Female: 11,103,346	20–84	Total: 21,592,374 NAFLD: 2,543,649 No NAFLD: 19,048,725	2009–2012	Age, sex, smoking status, alcohol consumption, regular physical exercise, income, diabetes mellitus, hypertension, and dyslipidemia
Kim et al. (2018)	2020	Korea	Male: 13,966 Female: 11,981	NAFLD: 50.1 ± 9.7 No NAFLD: 46.9 ± 10.2	Total: 25,947 NAFLD: 8,721 No NAFLD: 17,226	2004–2005	Demographic and metabolic factors
Wang et al. (2021)	2020	China	All male	NAFLD: 52.58 ± 12.07 No NAFLD: 53.12 ± 13.44	Total: 54,187 NAFLD: 8,721 No NAFLD: 36,659	2006–2007	Age, physical activity smoking status, body mass index, diabetes, hypertension, alcohol intake, education level, and serum levels of alanine
Lee et al. (2012)	2011	Korea	All female	35–80	Total: 5,517 NAFLD: 831 No NAFLD: 4,686	2002–2006	Age, smoking habits, and cardiometabolic risk factors
Wong et al. (2011)	2011	China	Male: 177 Female: 203	40–70	Total: 380 NAFLD: 199 No NAFLD: 181	2008–2010	Age and gender
Lin et al. (2014)	2014	China	Male: 1,370 Female: 945	NAFLD: 63.1 ± 12.8 (Male) 64.8 ± 11.5 (Female) No NAFLD:65.4 ± 13.8 (Male) 63.4 ± 14.3 (Female)	Total: 2,315 NAFLD: 263 No NAFLD: 2,052	2007–2011	Metabolic and other confounding factors
Lee et al. (2022)	2022	Korea	Male: 48.6% Female: 51.4%	40–64	Total: 8,933,017 NAFLD: 2,517,330 No NAFLD: 6,415,687	2009–2010	Age, sex, household income quartile, residential area Charlson Comorbidity Index, aspirin use, nonsteroidal anti-inflammatory drug use, tobacco
Cho et al. (2019)	2019	Korea	Male: 230 Female: 246	≥18	Total: 476 NAFLD: 379 No NAFLD: 97	2013–2018	Age‐, sex‐, the presence of hypertension and diabetes mellitus‐
Lee et al. (2020)	2020	Korea	Male: 4,234,418 Female: 3,886,256	≥20	Total: 812,0674 NAFLD: 936,159 No NAFLD: 7,184,515	2009–2017	Age, sex, smoking status, drinking habit, diabetes, regular exercise, yearly income, BMI
Stadlmayr et al. (2011)	2011	Austria	Male: 603 Female: 608	NAFLD: 62.1 ± 9.3 (Male) 62.61 ± 9.33 (Female) No NAFLD: 61.4 ± 10.3 (Male) 59.91 ± 10.89 (Female)	Total: 1,211 NAFLD: 632 No NAFLD: 579	2007–2009	Sex, age, BMI, liver steatosis, glucose intolerance
Hamaguchi et al. (2019)	2019	Japan	Male: 16,454 Female: 11,490	Male: 43.3 ± 8.2 Female: 44.7 ± 8.8	Total: 15,926 NAFLD: 3,211 No NAFLD: 12,715	2003–2016	Age, sex, smoking stutas, alcohol consumption, exercise and diabetes
Kim et al. (2021)	2021	Korea	Male: 4,659 Female: 1,523	NAFLD: 62.5 No NAFLD: 59.5	Total: 6,182 NAFLD: 2,643 No NAFLD: 3,540	2010–2017	NAFLD, age, current or former smoking, family history of colorectal cancer, regular exercise, use of nonsteroidal anti-inflammatory drugs, and baseline adenoma characteristics
Bhatt et al. (2015)	2015	United States	Male: 398 Female: 193	NAFLD: 62.5 No NAFLD: 59.5	Total: 591 NAFLD: 68 No NAFLD: 523	2008–2013	Age, NAFLD, and alcohol use
Ze et al. (2018)	2018	Korea	Male: 1,919 Female: 1,057	44–55	Total: 2,976 NAFLD: 1,512 No NAFLD: 1,464	2008–2013	—

We used NOS scale to evaluate the quality of included studies (Supplementary Table S1). Each study had a score larger or equal to seven and was thought to have a high quality. The average score of these studies were 8.357, which indicated an overall high quality of them.

### Non-alcoholic fatty liver disease and the risk of colorectal neoplasms

**FIGURE 2:**
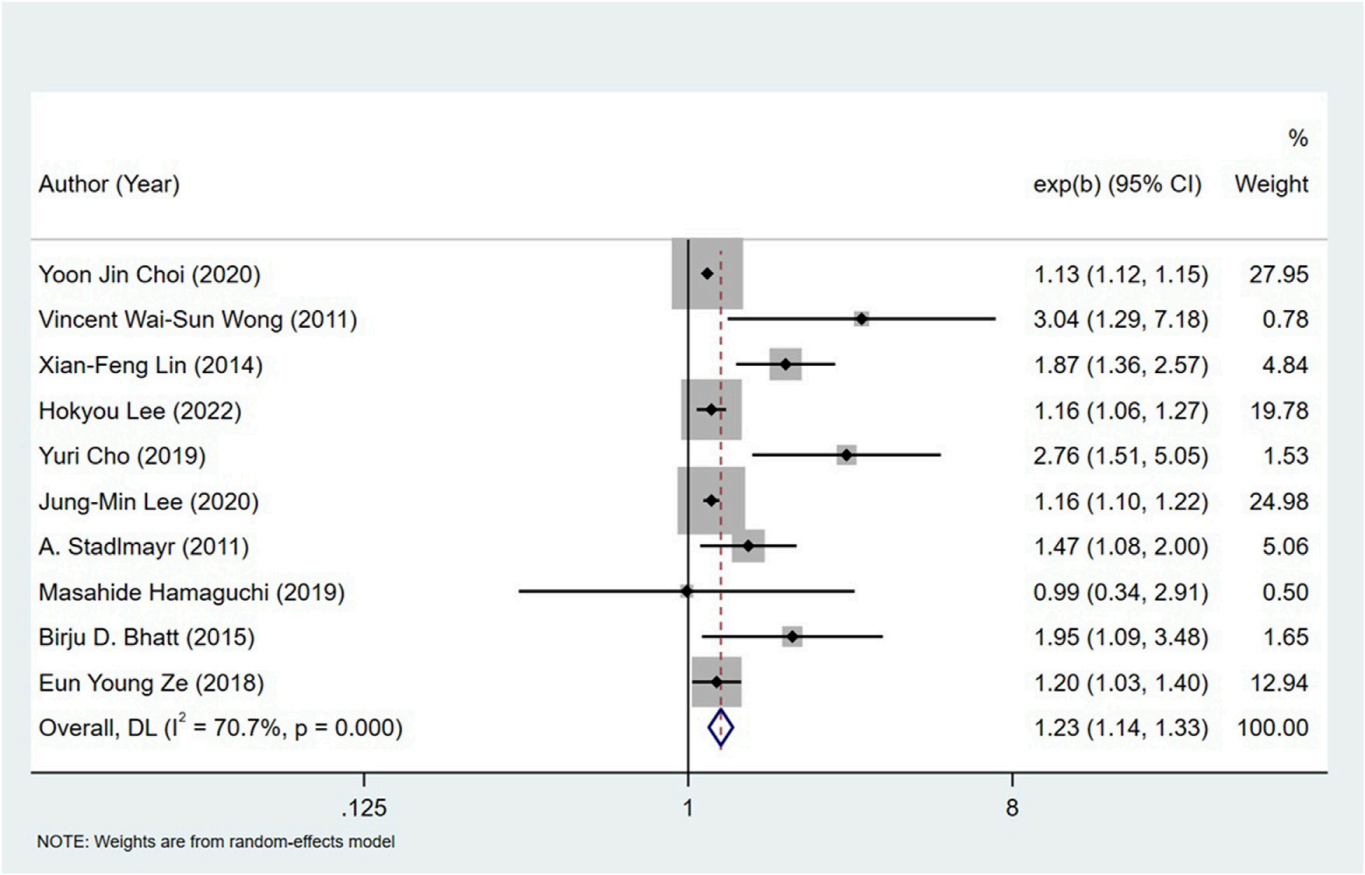
Forest plot showing the effect of NAFLD on CRC risk.

**FIGURE 3:**
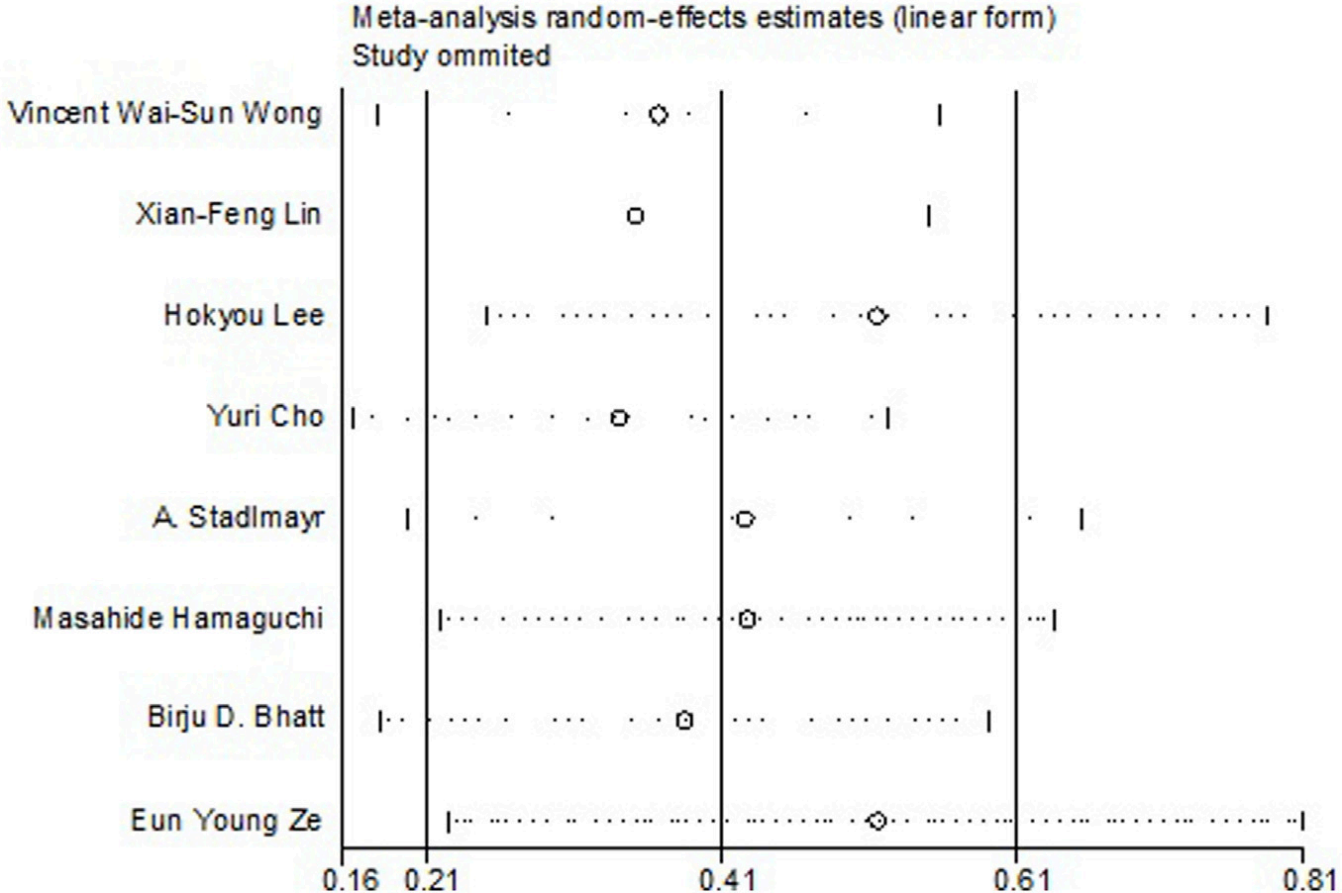
Sensitivity analysis showing the effect of NAFLD on CRC in eight literature.

**FIGURE 4:**
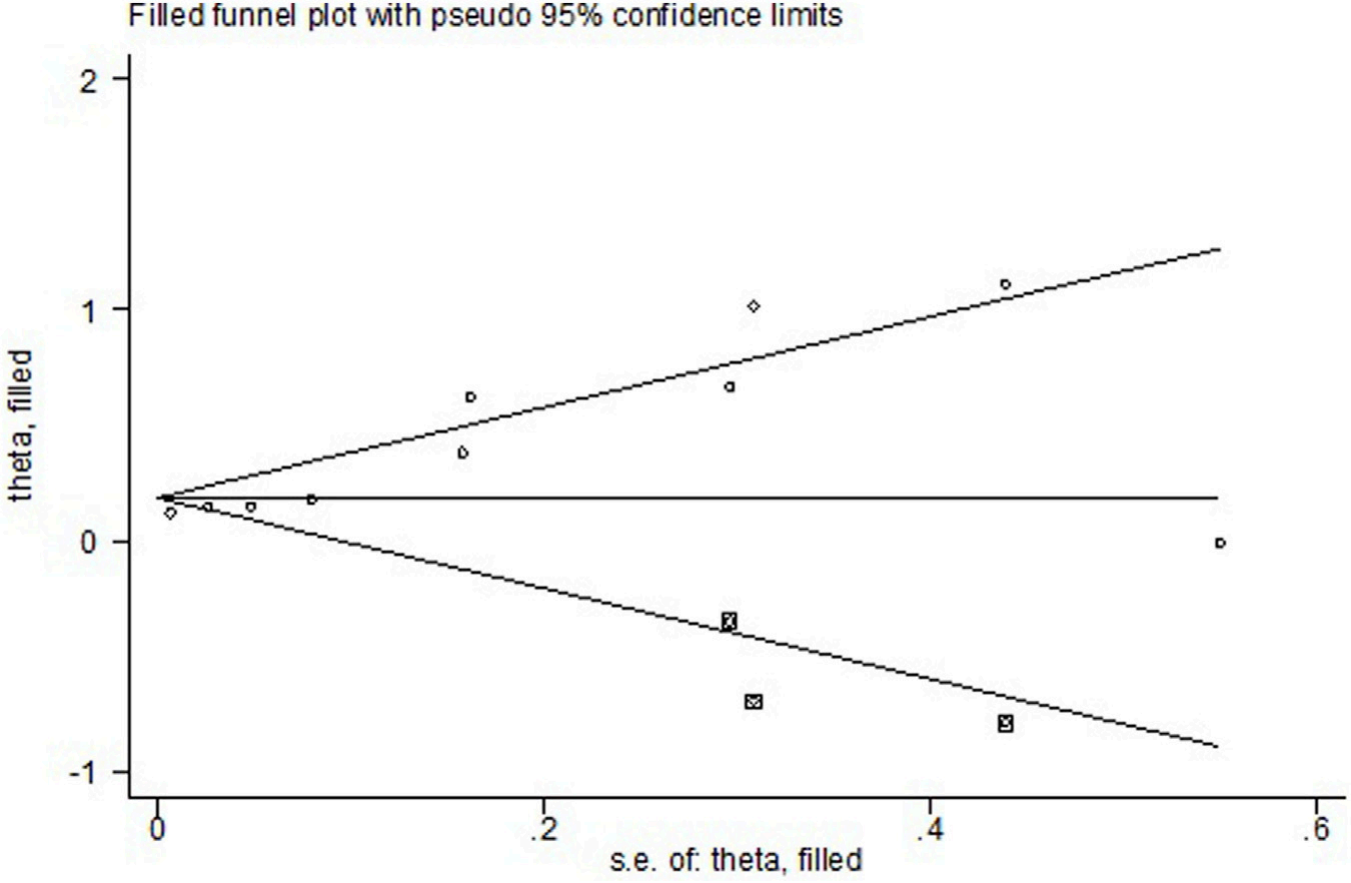
Publication bias of the risk of CRC caused by NAFLD.

All the 14 cohort studies explored the relationship between NAFLD and CRN, and all of them took CRN as outcome. Since four studies (Lee et al., 2012; Kim et al., 2018; Kim et al., 2021; Wang et al., 2021) respectively explored the relationship between NAFLD and CRA and CRC, they were excluded from the calculation of NAFLD and CRN, and were included in the subgroup study. The pooling analysis shows that NAFLD is associated with a higher risk of CRN (OR = 1.23; 95% CI: 1.14–1.33; I^2^ = 70.7%, *p* < 0.001; Figure 2). Due to the significant heterogeneity of the studies, sensitivity analysis was conducted and showed that 2 (Choi et al., 2020; Lee et al., 2020) of the 10 (Stadlmayr et al., 2011; Wong et al., 2011; Lin et al., 2014; Bhatt et al., 2015; Ze et al., 2018; Cho et al., 2019; Hamaguchi et al., 2019; Choi et al., 2020; Lee et al., 2020; Lee et al., 2022) articles may be the cause of the large heterogeneity. However, after removing the two literatures (Choi et al., 2020; Lee et al., 2020), the heterogeneity was almost the same as before, indicating that the two literatures were not the source of high heterogeneity (Figure 3). We used funnel plot and Egg’s test to verify the publication bias, and visual funnel plot was asymmetric. Bias was corrected by the trim and filling method. After that, no publication bias was found in the 13 literatures (Figure 4). Next, the 13 articles were combined (OR = 3.34, 95% CI: 3.03–3.73, *p* < 0.001). The results before and after the trim and filling method were compared. The correlation between NAFLD and CRN was slightly increased after this adjustment, which could be considered as stable results of the existing meta-analysis (Supplementary Figures S1, S2).

### Non-alcoholic fatty liver disease and the risk of colorectal adenoma and colorectal cancer

We separately analyzed CRA and CRC in the subgroup study. Seven studies (Stadlmayr et al., 2011; Wong et al., 2011; Lee et al., 2012; Lin et al., 2014; Bhatt et al., 2015; Ze et al., 2018; Cho et al., 2019) explored the relationship between NAFLD and CRA, concluding that NAFLD increased the risk of CRA (OR = 1.29; 95% CI = 1.15–1.45; I^2^ =66.4%, *p* > 0.001). 10 studies (Wong et al., 2011; Lee et al., 2012; Lin et al., 2014; Kim et al., 2018; Cho et al., 2019; Hamaguchi et al., 2019; Choi et al., 2020; Lee et al., 2020; Wang et al., 2021; Lee et al., 2022) investigated the relationship between NAFLD and CRC, reflecting that NAFLD augments the risk of CRC (OR = 1.13; 95% CI =1.12–1.15; I^2^ = 69.4%, *p* < 0.001).

### Gender and smoking of non-alcoholic fatty liver disease patients and the risk of colorectal neoplasms

**FIGURE 5:**
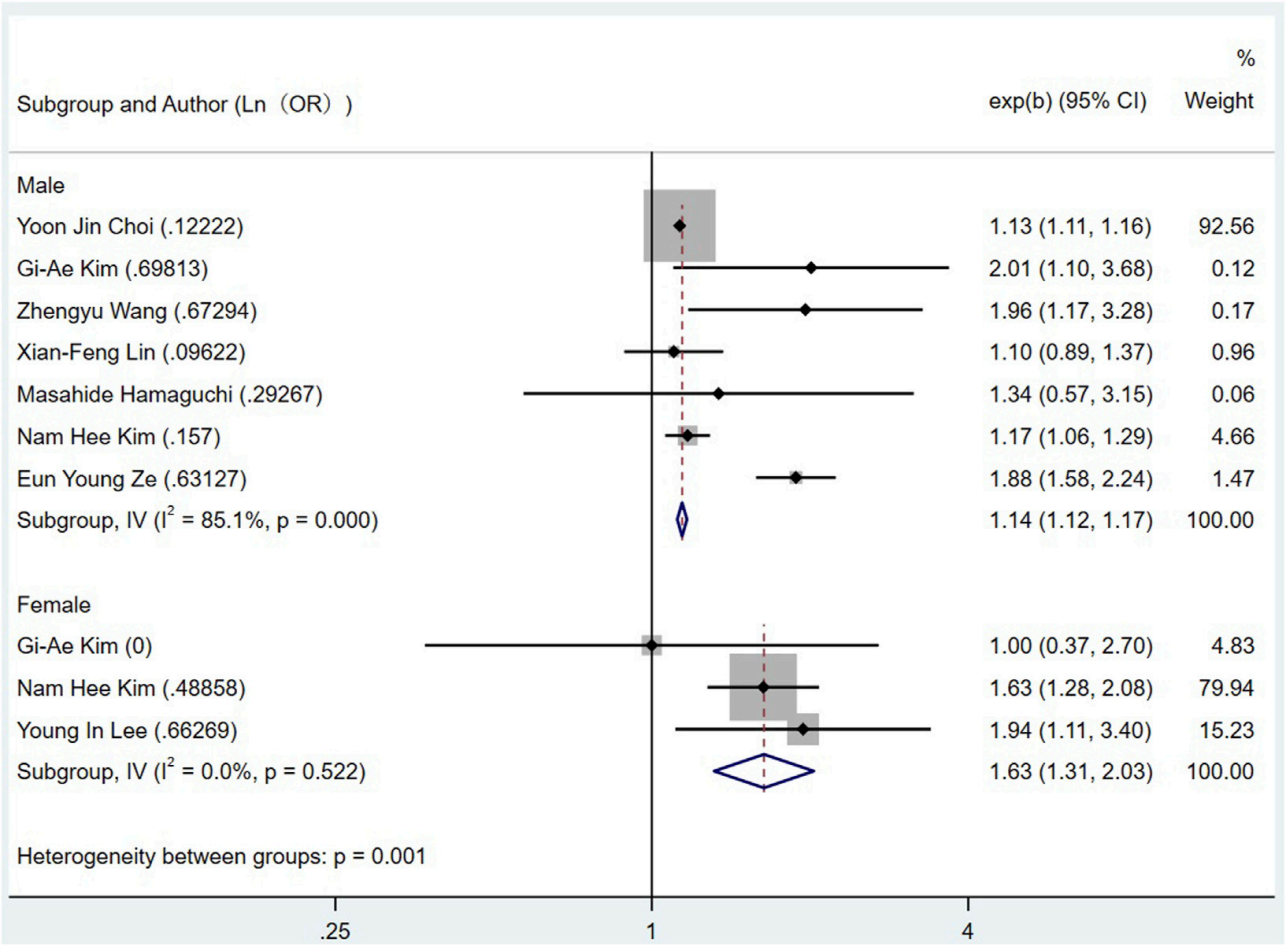
Subgroup analysis for the risk of CRC in patients with NAFLD in different genders.

Eight literatures (Lee et al., 2012; Lin et al., 2014; Kim et al., 2018; Ze et al., 2018; Hamaguchi et al., 2019; Choi et al., 2020; Kim et al., 2021; Wang et al., 2021) studied the correlation between the gender of NAFLD patients and the risk of CRN, and found that the OR of male NAFLD patients with CRN was 1.14 (95% CI = 1.12–1.17; I^2^ = 85.1%, *p* < 0.001), and that of female patients was 1.63 (95% CI = 1.31–2.03; I^2^ = 0%, *p* = 0.522) (Figure 5). The size of samples and the amounts of researches need to be expanded to get a more accurate result about this issue.

**FIGURE 6:**
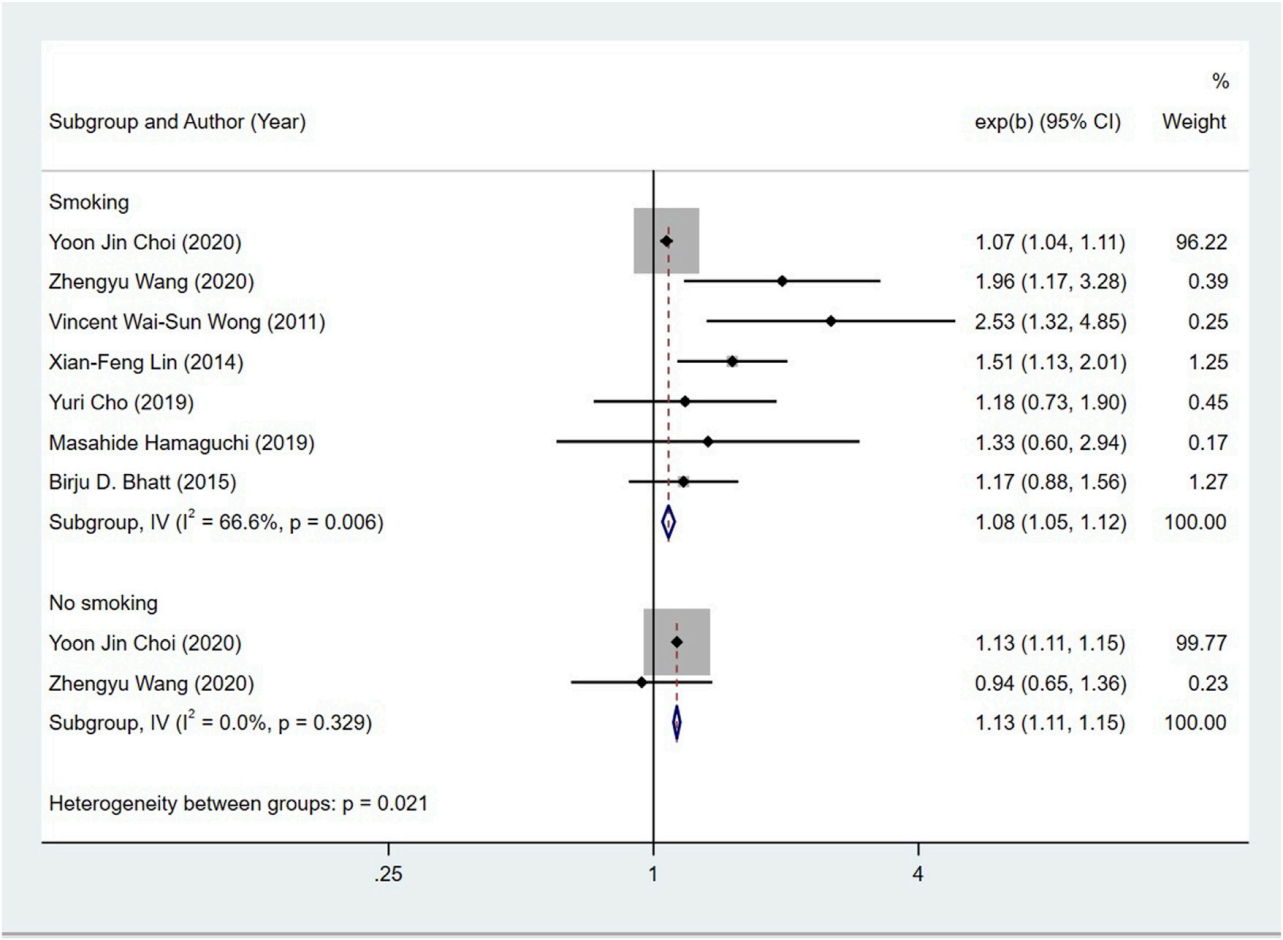
Subgroup analysis for the risk of CRC in patients with NAFLD in different smoking statuses.

Seven studies (Wong et al., 2011; Lin et al., 2014; Bhatt et al., 2015; Cho et al., 2019; Hamaguchi et al., 2019; Choi et al., 2020; Wang et al., 2021) examined the association between the NAFLD patients in smoking and their risk of CRN, and the OR was 1.08 (95% CI = 1.05–1.12; I^2^ = 66.6%, *p* > 0.001). Two studies (Choi et al., 2020; Wang et al., 2021) investigated the relationship between non-smoking NAFLD patients and the risk of CRN, and the OR was 1.13 (95% CI = 1.11–1.15, I^2^ = 0%, *p* > 0.001). Given this, there is no close correlation between smoking status of NAFLD patients and CRN risk (Figure 6).

### Ferroptosis and cuproptosis may link non-alcoholic fatty liver disease with colorectal neoplasms

**FIGURE 7:**
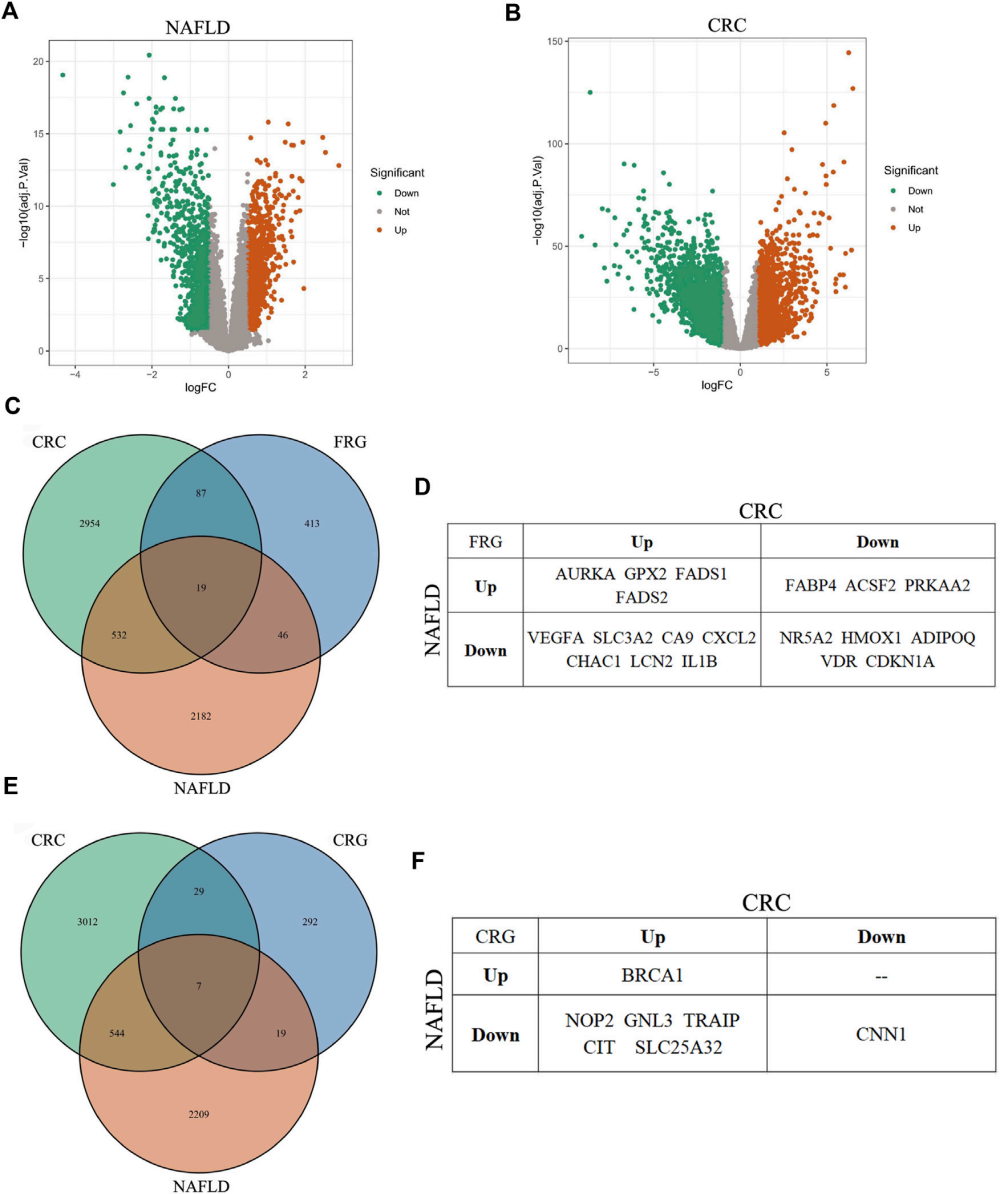
Analysis of DEGs in NAFLD, CRC, ferroptosis, and cuproptosis. **(A,B)** Volcano plot of DEGs in NAFLD and CRC, respectively. **(C)** Venn diagram of overlapping DEGs among NAFLD, CRC, and ferroptosis, respectively. **(D)** The overlapping DEGs among all the three gene sets: NAFLD, CRC, and ferroptosis. **(E)** Venn diagram of overlapping DEGs among NAFLD, CRC, and cuproptosis, respectively. **(F)** The overlapping DEGs among all the three gene sets: NAFLD, CRC, and cuproptosis.

We identified DEGs from GSE89632 dataset for NAFLD and TCGA-COAD dataset for CRC, respectively. In NAFLD, 2,779 DEGs were identified: 1,252 genes were upregulated and 1,527 genes were downregulated (Figure 7A). In CRC, 3,592 DEGs were identified: 1,426 genes were upregulated and 2,166 genes were downregulated (Figure 7B).

Ferroptosis is a novel iron-dependent cell death type, which is closely relevant to various disorders, including NAFLD and CRN (Stockwell, 2022). However, whether ferroptosis has a role in NAFLD-caused CRN or not, is currently elusive. Here we performed bioinformatics analysis to get some clues about this question. We focused on CRC, mainly basing on two reasons. One is that CRC is a more malignant tumor type than CRA, which needs more clinical and scientific attention. The other reason is that the relationship between ferroptosis and CRC has been much more detailedly studied compared with that between ferroptosis and CRA. The DEG overlap analysis among the three gene datasets, including the DEGs for NAFLD, CRC, and ferroptosis-related genes (FRGs), identified 19 genes (Figures 7C,D; Supplementary Table S3).

Cuproptosis was coined in 2022 (Tsvetkov et al., 2022). This term represents a quite novel cell death mode. Although there are not yet too many researches about cuproptosis, its major mechanism implies that cuproptosis is involved in the pathology of diverse diseases, just like ferroptosis. Then we determined whether there were overlap genes among NAFLD, CRC, and cuproptosis. Similar to the analysis of ferroptosis, the overlap DEGs among the other three gene datasets, including the DEGs for NAFLD, CRC, and cuproptosis-related genes (CRGs), contained seven genes (Figures 7E,F; Supplementary Table S4). These results suggested that ferroptosis and cuproptosis might have a role in NAFLD-related CRC, severally (see following detailed discussion).

## Discussion

As the most common chronic liver disease around the world, NAFLD is diagnosed by the accumulation of fat in the liver without long-term drinking or drinking enough alcohol with a history of 40 g/day (Dumitrascu and Neuman, 2018). The occurrence of NAFLD is related to many factors, such as MetS, obesity, type 2 diabetes mellitus, insulin resistance, dyslipidemia, and gender (Sweet et al., 2017). Lifestyle factors, such as a high-fat diet, a high-fructose diet, consuming a lot of processed red meat, and being sedentary, can cause metabolic changes in the body, leading to insulin resistance and dysbiosis of the gut microbiota, which may finally result in NAFLD. CRN (particularly CRC) is now a severe healthy problem in both developing and developed regions. In addition to age and genetic factors, familial adenomatous polyposis and environmental factors including physical activity and diet habit (drinking alcohol, smoking, and excessive red meat consumption) are major risk factors for CRC (Haggar and Boushey, 2009).

Since NAFLD and CRN are both lifestyle-related diseases, the relationship between NAFLD and CRN risk is biologically plausible. This study includes two parts: 1) meta-analysis of existing literatures to explore the correlation between NAFLD and CRN; and 2) bioinformatics analysis to interrogate the role of ferroptosis and cuproptosis in NAFLD-related CRN. The meta-analysis incorporated 14 cohort studies, covering 38,761,773 individuals, to reveal the relationship between NAFLD and CRN in detail. Noteworthily, we included all high-quality cohort studies, complemented recent studies, and performed relevant subgroup studies. We found that NAFLD patients had an upregulated risk of getting CRN, with an average of 1.23-fold increase compared with patients without NAFLD. This suggests that NAFLD needs to be treated as a new risk factor for CRN.

In CRN subgroup studies, the risk of CRA in patients with NAFLD was 1.29 times higher than that in patients without NAFLD, and this parameter for CRC was 1.13 times. Since CRA is a precursor of CRC, the role of NAFLD in both CRA and CRC cannot be ignored. In gender subgroup, the risk of CRN in male and female NAFLD patients was 1.14 and 1.63 times higher than that in non-NAFLD patients, respectively. Notably, previous studies have concluded that male patients with NAFLD are more likely to have CRN than female patients. This inconsistency may be due to the lack of female-based risk studies in our project, resulting in inaccurate results. The calculation result *p* = 0.522 > 0.01 can support this possibility. More samples and parameters are needed to get a reliable conclusion about this issue. In the smoking subgroup, the OR of CRN in NAFLD patients who smoked was 1.08, and that in those who did not smoke was 1.13, indicating that smoking status had no significant effect on whether NAFLD patients would have CRN.

About the underlying causes that link NAFLD with CRN, researchers now hold several mechanisms in mind. First, NAFLD can change the systemic immune state and upregulate the level of various pro-tumor cytokines, which promotes the initiation and development of CRN (Kim et al., 2008; Hwang et al., 2010). Second, NAFLD can cause insulin resistance and augment the amount of circulating insulin. Insulin has been implicated as an oncogenic factor in CRC. In addition, elevated plasma glucose and insulin levels, obesity, and physical inactivity are also related to colorectal cancer (Basyigit et al., 2015). Third, NAFLD results in upregulated fatty acids and carbohydrates levels in blood, which can fuel the development of CRN (Hirsova and Ibrahim, 2017). Fourth, during the development of NAFLD, the low inflammatory state caused by TNF-α and IL-6 leads to the disorder of gut microbiota, which can also initiate colorectal cancer (Boursier et al., 2016). These factors caused by NAFLD contribute together to the pathology of CRN. Targeting these mechanisms could help control NAFLD-related CRN. However, other links between NAFLD and CRN are also warranted to be discovered.

Ferroptosis and cuproptosis are two newly identified cell death types. Ferroptosis (which is majorly modulated by p53 and GPX4) participates in many disorders, while the pathological relevance of cuproptosis has not be fully studied (Liu et al., 2019; Liu and Gu, 2021; Liu and Gu, 2022; Stockwell, 2022; Tsvetkov et al., 2022). To date, the relationship between ferroptosis/cuproptosis and NAFLD remains largely unknown. Given this, we leveraged the online databases to perform bioinformatics analysis and identified many deregulated ferroptosis- and cuproptosis-related genes in NAFLD, respectively (Figures 7A,C,E). This result suggested that ferroptosis and cuproptosis might involve the pathology of NAFLD. Interestingly, ferroptosis and cuproptosis are both associated with CRN (especially CRC) (Li et al., 2022; Zhang et al., 2022). This finding urged us to pursue the potential significance of ferroptosis and cuproptosis in NAFLD-related CRN (here we use CRC as an example), respectively. For ferroptosis, we compared the differentially expressed genes among NAFLD, CRC, and ferroptosis. We indeed identified 19 overlap genes among the three categories, such as GPX2, CDKN1A, SLC3A2, and VEGFA (Figures 7C,D; Supplementary Table S3). These shared genes may hint a possible role of ferroptosis in NAFLD-related CRC. Carefully analyzing this overlap gene set, we can conclude at least three scientific or clinical potentials of it. First, these genes may represent certain unknown mechanistic link between NAFLD and CRC. These genes in NAFLD may somehow affect the expression of the same gene set in CRC. A more conceivable possibility is that the two altered ferroptosis-related gene sets may result from a common upstream regulator or factor, for example, MetS, given these two diseases share a lot of risk factors. Second, these genes may dictate the choice of therapeutics for CRC patients who get NAFLD. For example, GPX2 is upregulated in both NAFLD and CRC. A systemic GPX2 inhibitor may alleviate NAFLD, and at the same time induce ferroptosis to kill CRC cells. However, SLC3A2 is downregulated in NAFLD, but CRC increases its expression to ensure the biogenesis of GSH to suppress ferroptosis. Due to this inconsistency, when choosing drugs targeting SLC3A2 to treat CRC, there is a caveat to consider the risk to exacerbate NAFLD. Tumor-specific drug delivery will be a good choice for this situation. Taken together, the overlap gene set can give the clinicians invaluable information when considering which gene should be targeted to treat CRC when the patient also has NAFLD. With the help of this gene set, a dual positive effects or a less negative effect could be achieved in the treatment of these patients. Third, FRGs have prognostic value in CRC (Hu et al., 2022). According to our results, an inner correlation between FRG in NAFLD and those in CRC exists. The differentially-expressed FRG set in NAFLD may predict the survival of the same patient who is also suffering from CRC. This gene set can be used as auxiliary evidence for CRC prognosis based on the information from the bulk tumor tissue. For those patients who are only with NAFLD, but have not yet been diagnosed with CRC, this gene set from NAFLD could predict the risk of these patients to develop a malignant or a benign CRN, which is good for them to prevent the occurrence of CRN. Although research about cuproptosis is just getting started, the logic behind the role of ferroptosis in NAFLD-related CRN also applies to cuproptosis (Figures 7E,F; Supplementary Table S4) (Liu et al., 2022; Xu et al., 2022). In conclusion, ferroptosis and cuproptosis may have important roles in NAFLD-related CRN, not only in basic scientific research, but also in clinical application.

It is recommended that people over 50 years old should undergo routine colonoscopy every year, which is an effective way to reduce the incidence and mortality of colorectal neoplasms (Zauber et al., 2012; Nishihara et al., 2013). Based on the conclusion of this study, we should put more emphasis on calling for colonoscopy in patients with NAFLD, regardless of age and gender, to minimize the risk of CRN. To achieve this, popular medical science propaganda should be performed, particularly aiming at the population with NAFLD, or risk factors associated with NAFLD and CRN. More responsibilities should be laid on the public health policy makers to optimize these effective means. For clinicians, our results provide invaluable information for the development of new diagnostic and therapeutic methods targeting NAFLD-related CRN.

This study also has some limitations. Case-control and cross-sectional studies can be included in future studies to improve the conclusion. In addition, covariate analysis was not included in this current project. However, the studies included were controlled for adjusted confounding factors, making the conclusion in our study reliable and easy to be translated into clinical practice. Another issue is the heterogeneity existing in these studies, which was large and could not be decreased after removing each article and recalculating and subgroup analysis. Diagnostic criteria are not identical across regions and some studies relied primarily on electronic health record correlation for diagnosis, which may contribute to the high heterogeneity. Some literatures used global CRN as the outcome, while some used CRA or CRC alone as the outcome, which may also lead to the high heterogeneity. Moreover, confounders in each study could also result in heterogeneity. More systematic and standardized studies are needed to lessen the heterogeneity and consolidate our conclusion.

To summarize, this meta- and bioinformatics analysis suggests that NAFLD increases the risk of CRN. For the first time we revealed that ferroptosis and cuproptosis may link NAFLD with CRN, respectively. The results in our study can be quite effective in developing CRN prevention, diagnosis, and treatment strategies.

## Data Availability

The original contributions presented in the study are included in the article/Supplementary Material, further inquiries can be directed to the corresponding authors.

## Supplementary material

SUPPLEMENTARY FIGURE S1 Sensitivity analysis showing the effect of NAFLD on CRC in 10 literature.

SUPPLEMENTARY FIGURE S2 Funnel figure showing the effect of NAFLD on CRC.

## Abbreviations

NAFLD, Non-alcoholic fatty liver disease; CRN, Colorectal neoplasm; CRA, Colorectal adenoma; CRC, Colorectal cancer; MetS, Metabolic syndrome; MeSH, Medical Subject Headings; OR, Odds ratio; CI, Confidence interval; NOS, Newcastle-Ottawa scale; DEG, Differentially expressed gene; FRG, Ferroptosis-related gene; CRG, Cuproptosis-related gene.

## References

[B1] Armstrong M. J. Adams L. A. Canbay A. Syn W. K. (2014). Extrahepatic complications of nonalcoholic fatty liver disease. Hepatology 59 (3), 1174–1197. 10.1002/hep.26717 24002776

[B2] Assy N. Kaita K. Mymin D. Levy C. Rosser B. Minuk G. (2000). Fatty infiltration of liver in hyperlipidemic patients. Dig. Dis. Sci. 45 (10), 1929–1934. 10.1023/a:1005661516165 11117562

[B3] Basyigit S. Uzman M. Kefeli A. Sapmaz F. P. Yeniova A. O. Nazligul Y. (2015). Absence of non-alcoholic fatty liver disease in the presence of insulin resistance is a strong predictor for colorectal carcinoma. Int. J. Clin. Exp. Med. 8 (10), 18601–18610. 26770473 PMC4694373

[B4] Bhatt B. D. Lukose T. Siegel A. B. Brown R. S. Jr. Verna E. C. (2015). Increased risk of colorectal polyps in patients with non-alcoholic fatty liver disease undergoing liver transplant evaluation. J. Gastrointest. Oncol. 6 (5), 459–468. 10.3978/j.issn.2078-6891.2015.050 26487938 PMC4570908

[B5] Boursier J. Mueller O. Barret M. Machado M. Fizanne L. Araujo-Perez F. (2016). The severity of nonalcoholic fatty liver disease is associated with gut dysbiosis and shift in the metabolic function of the gut microbiota. Hepatology 63 (3), 764–775. 10.1002/hep.28356 26600078 PMC4975935

[B6] Budhathoki S. Iwasaki M. Yamaji T. Sasazuki S. Takachi R. Sakamoto H. (2015). Dietary heterocyclic amine intake, NAT2 genetic polymorphism, and colorectal adenoma risk: The colorectal adenoma study in tokyo. Cancer Epidemiol. Biomarkers Prev. 24 (3), 613–620. 10.1158/1055-9965.EPI-14-1051 25604583

[B7] Byrne C. D. Targher G. (2015). Nafld: A multisystem disease. J. Hepatol. 62, S47–S64. 10.1016/j.jhep.2014.12.012 25920090

[B8] Chacko K. R. Reinus J. (2016). Extrahepatic complications of nonalcoholic fatty liver disease. Clin. Liver Dis. 20 (2), 387–401. 10.1016/j.cld.2015.10.004 27063276

[B9] Cho Y. Lim S. K. Joo S. K. Jeong D. H. Kim J. H. Bae J. M. (2019). Nonalcoholic steatohepatitis is associated with a higher risk of advanced colorectal neoplasm. Liver Int. 39 (9), 1722–1731. 10.1111/liv.14163 31162812

[B10] Choi Y. J. Lee D. H. Han K. D. (2020). Association between high fatty liver index and development of colorectal cancer: A nationwide cohort study with 21, 592, 374 Korean. Korean J. Intern. Med. 35 (6), 1354–1363. 10.3904/kjim.2018.022 32264657 PMC7652640

[B11] Donati G. Stagni B. Piscaglia F. Venturoli N. Morselli-Labate A. M. Rasciti L. (2004). Increased prevalence of fatty liver in arterial hypertensive patients with normal liver enzymes: Role of insulin resistance. Gut 53 (7), 1020–1023. 10.1136/gut.2003.027086 15194655 PMC1774102

[B12] Dumitrascu D. L. Neuman M. G. (2018). Non-alcoholic fatty liver disease: An update on diagnosis. Clujul Med. 91 (2), 147–150. 10.15386/cjmed-993 29785151 PMC5958978

[B13] Haggar F. A. Boushey R. P. (2009). Colorectal cancer epidemiology: Incidence, mortality, survival, and risk factors. Clin. Colon Rectal Surg. 22 (4), 191–197. 10.1055/s-0029-1242458 21037809 PMC2796096

[B14] Hamaguchi M. Hashimoto Y. Obora A. Kojima T. Fukui M. (2019). Non-alcoholic fatty liver disease with obesity as an independent predictor for incident gastric and colorectal cancer: A population-based longitudinal study. BMJ Open Gastroenterol. 6 (1), e000295. 10.1136/bmjgast-2019-000295 PMC6577367 31275587

[B15] Hirsova P. Ibrahim S. H. (2017). Lipotoxic lethal and sublethal stress signaling in hepatocytes: Relevance to NASH pathogenesis (vol 57, pg 1758, 2016). J. Lipid Res. 58 (1), 299. 27049024 10.1194/jlr.R066357 PMC5036373

[B16] Hu D. Zhou Z. Wang J. Zhu K. (2022). Screening of ferroptosis-related genes with prognostic effect in colorectal cancer by bioinformatic analysis. Front. Mol. Biosci. 9, 979854. 10.3389/fmolb.2022.979854 36203871 PMC9531163

[B17] Hwang S. T. Cho Y. K. Park J. H. Kim H. J. Park D. I. Sohn C. I. (2010). Relationship of non-alcoholic fatty liver disease to colorectal adenomatous polyps. J. Gastroenterol. Hepatol. 25 (3), 562–567. 10.1111/j.1440-1746.2009.06117.x 20074156

[B18] Kim G. A. Lee H. C. Choe J. Kim M. J. Lee M. J. Chang H. S. (2018). Association between non-alcoholic fatty liver disease and cancer incidence rate. J. hepatology 68 (1), 140–146. 10.1016/j.jhep.2017.09.012 29150142

[B19] Kim N. H. Jung Y. S. Park J. H. Park D. I. Sohn C. I. (2021). Impact of nonalcoholic fatty liver disease on the risk of metachronous colorectal neoplasia after polypectomy. Korean J. Intern. Med. 36 (3), 557–567. 10.3904/kjim.2019.360 32630984 PMC8137416

[B20] Kim S. Keku T. O. Martin C. Galanko J. Woosley J. T. Schroeder J. C. (2008). Circulating levels of inflammatory cytokines and risk of colorectal adenomas. Cancer Res. 68 (1), 323–328. 10.1158/0008-5472.CAN-07-2924 18172326 PMC2675825

[B21] Ladabaum U. Dominitz J. A. Kahi C. Schoen R. E. (2020). Strategies for colorectal cancer screening. Gastroenterology 158 (2), 418–432. 10.1053/j.gastro.2019.06.043 31394083

[B22] Lee H. Lee H. W. Kim S. U. Kim H. C. (2022). Metabolic dysfunction-associated fatty liver disease increases colon cancer risk: A nationwide cohort study. Clin. Transl. Gastroenterol. 13 (1), e00435. 10.14309/ctg.0000000000000435 35080508 PMC8806363

[B23] Lee J. M. Park Y. M. Yun J. S. Ahn Y. B. Lee K. M. Kim D. B. (2020). The association between nonalcoholic fatty liver disease and esophageal, stomach, or colorectal cancer: National population-based cohort study. PloS one 15 (1), e0226351. 10.1371/journal.pone.0226351 31978054 PMC6980645

[B24] Lee Y. I. Lim Y. S. Park H. S. (2012). Colorectal neoplasms in relation to non-alcoholic fatty liver disease in Korean women: A retrospective cohort study. J. Gastroenterol. Hepatol. 27 (1), 91–95. 10.1111/j.1440-1746.2011.06816.x 21679251

[B25] Li Y. Wang R. Y. Deng Y. J. Wu S. H. Sun X. Mu H. (2022). Molecular characteristics, clinical significance, and cancer immune interactions of cuproptosis and ferroptosis-associated genes in colorectal cancer. Front. Oncol. 12, 975859. 10.3389/fonc.2022.975859 36132144 PMC9483209

[B26] Lin X. F. Shi K. Q. You J. Liu W. Y. Luo Y. W. Wu F. L. (2014). Increased risk of colorectal malignant neoplasm in patients with nonalcoholic fatty liver disease: A large study. Mol. Biol. Rep. 41 (5), 2989–2997. 10.1007/s11033-014-3157-y 24449368

[B27] Liu Y. Gu W. (2022). p53 in ferroptosis regulation: the new weapon for the old guardian. Cell Death Differ. 29 (5), 895–910. 10.1038/s41418-022-00943-y 35087226 PMC9091200

[B28] Liu Y. Gu W. (2021). The complexity of p53-mediated metabolic regulation in tumor suppression. Semin. Cancer Biol. 85, 4–32. 10.1016/j.semcancer.2021.03.010 33785447 PMC8473587

[B29] Liu Y. Q. Liu Y. Ye S. J. Feng H. J. Ma L. J. (2022). Development and validation of cuproptosis-related gene signature in the prognostic prediction of liver cancer. Front. Oncol. 12, 985484. 10.3389/fonc.2022.985484 36033443 PMC9413147

[B30] Liu Y. Q. Tavana O. Gu W. (2019). p53 modifications: exquisite decorations of the powerful guardian. J. Mol. Cell Biol. 11 (7), 564–577. 10.1093/jmcb/mjz060 31282934 PMC6736412

[B31] Nishihara R. Wu K. N. Lochhead P. Morikawa T. Liao X. Y. Qian Z. R. (2013). Long-term colorectal-cancer incidence and mortality after lower endoscopy. N. Engl. J. Med. 369 (12), 1095–1105. 10.1056/NEJMoa1301969 24047059 PMC3840160

[B32] Page M. J. McKenzie J. E. Bossuyt P. M. Boutron I. Hoffmann T. C. Mulrow C. D. (2021). The PRISMA 2020 statement: An updated guideline for reporting systematic reviews. J. Clin. Epidemiol. 134, 178–189. 10.1016/j.jclinepi.2021.03.001 33789819

[B33] Sclafani F. Gullo G. Sheahan K. Crown J. (2013). BRAF mutations in melanoma and colorectal cancer: A single oncogenic mutation with different tumour phenotypes and clinical implications. Crit. Rev. Oncol. Hematol. 87 (1), 55–68. 10.1016/j.critrevonc.2012.11.003 23246082

[B34] Stadlmayr A. Aigner E. Steger B. Scharinger L. Lederer D. Mayr A. (2011). Nonalcoholic fatty liver disease: An independent risk factor for colorectal neoplasia. J. Intern. Med. 270 (1), 41–49. 10.1111/j.1365-2796.2011.02377.x 21414047

[B35] Stang A. (2010). Critical evaluation of the Newcastle-Ottawa scale for the assessment of the quality of nonrandomized studies in meta-analyses. Eur. J. Epidemiol. 25 (9), 603–605. 10.1007/s10654-010-9491-z 20652370

[B36] Stockwell B. R. (2022). Ferroptosis turns 10: Emerging mechanisms, physiological functions, and therapeutic applications. Cell 185 (14), 2401–2421. 10.1016/j.cell.2022.06.003 35803244 PMC9273022

[B37] Sung H. Ferlay J. Siegel R. L. Laversanne M. Soerjomataram I. Jemal A. (2021). Global cancer statistics 2020: GLOBOCAN estimates of incidence and mortality worldwide for 36 cancers in 185 countries. Ca. Cancer J. Clin. 71 (3), 209–249. 10.3322/caac.21660 33538338

[B38] Sweet P. H. Khoo T. Nguyen S. (2017). Nonalcoholic fatty liver disease. Prim. Care 44 (4), 599–607. 10.1016/j.pop.2017.07.003 29132522

[B39] Taylor K. S. Mahtani K. R. Aronson J. K. (2021). Summarising good practice guidelines for data extraction for systematic reviews and meta-analysis. BMJ Evid. Based. Med. 26 (3), 88–90. 10.1136/bmjebm-2020-111651 33632720

[B40] Tsvetkov P. Coy S. Petrova B. Dreishpoon M. Verma A. Abdusamad M. (2022). Copper induces cell death by targeting lipoylated TCA cycle proteins. Science 375 (6586), 1254–1261. 10.1126/science.abf0529 35298263 PMC9273333

[B41] Wang Z. Y. Zhao X. Y. Chen S. H. Wang Y. H. Cao L. Y. Liao W. (2021). Associations between nonalcoholic fatty liver disease and cancers in a large cohort in China. Clin. Gastroenterol. Hepatol. 19 (4), 788–796.e4. 10.1016/j.cgh.2020.05.009 32407969

[B42] Wong V. W. S. Wong G. L. H. Tsang S. W. C. Fan T. Chu W. C. W. Woo J. (2011). High prevalence of colorectal neoplasm in patients with non-alcoholic steatohepatitis. Gut 60 (6), 829–836. 10.1136/gut.2011.237974 21339204

[B43] Xu M. R. Mu J. Y. Wang J. J. Zhou Q. Wang J. W. (2022). Construction and validation of a cuproptosis-related lncRNA signature as a novel and robust prognostic model for colon adenocarcinoma. Front. Oncol. 12, 961213. 10.3389/fonc.2022.961213 35965536 PMC9367690

[B44] Younossi Z. M. Koenig A. B. Abdelatif D. Fazel Y. Henry L. Wymer M. (2016). Global epidemiology of nonalcoholic fatty liver disease-meta-analytic assessment of prevalence, incidence, and outcomes. Hepatology 64 (1), 73–84. 10.1002/hep.28431 26707365

[B45] Zauber A. G. Winawer S. J. O'Brien M. J. Lansdorp-Vogelaar I. van Ballegooijen M. Hankey B. F. (2012). Colonoscopic polypectomy and long-term prevention of colorectal-cancer deaths. N. Engl. J. Med. 366 (8), 687–696. 10.1056/NEJMoa1100370 22356322 PMC3322371

[B46] Ze E. Y. Kim B. J. Jun D. H. Kim J. G. Kang H. Lee D. Y. (2018). The fatty liver index: A simple and accurate predictor of colorectal adenoma in an average-risk population. Dis. Colon Rectum 61 (1), 36–42. 10.1097/DCR.0000000000000973 29219920 PMC5728585

[B47] Zhang Q. M. Deng T. Zhang H. D. Zuo D. Zhu Q. H. Bai M. (2022). Adipocyte-derived exosomal MTTP suppresses ferroptosis and promotes chemoresistance in colorectal cancer. Adv. Sci. 9, e2203357. 10.1002/advs.202203357 PMC9534973 35978266

