# Supplementary figures and images for: Analysis of the correlation between non-alcoholic fatty liver disease and the risk of colorectal neoplasms
Source: Front Pharmacol. 2022 Nov 9;13:1068432. doi: 10.3389/fphar.2022.1068432 (PMC9682006; doi:10.3389/fphar.2022.1068432)

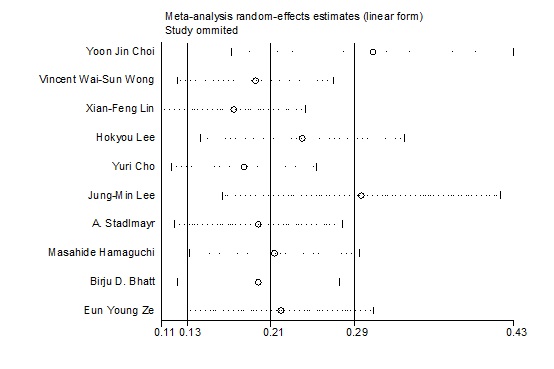

Supplement: Supplementary file 2 [file Image1.JPEG]

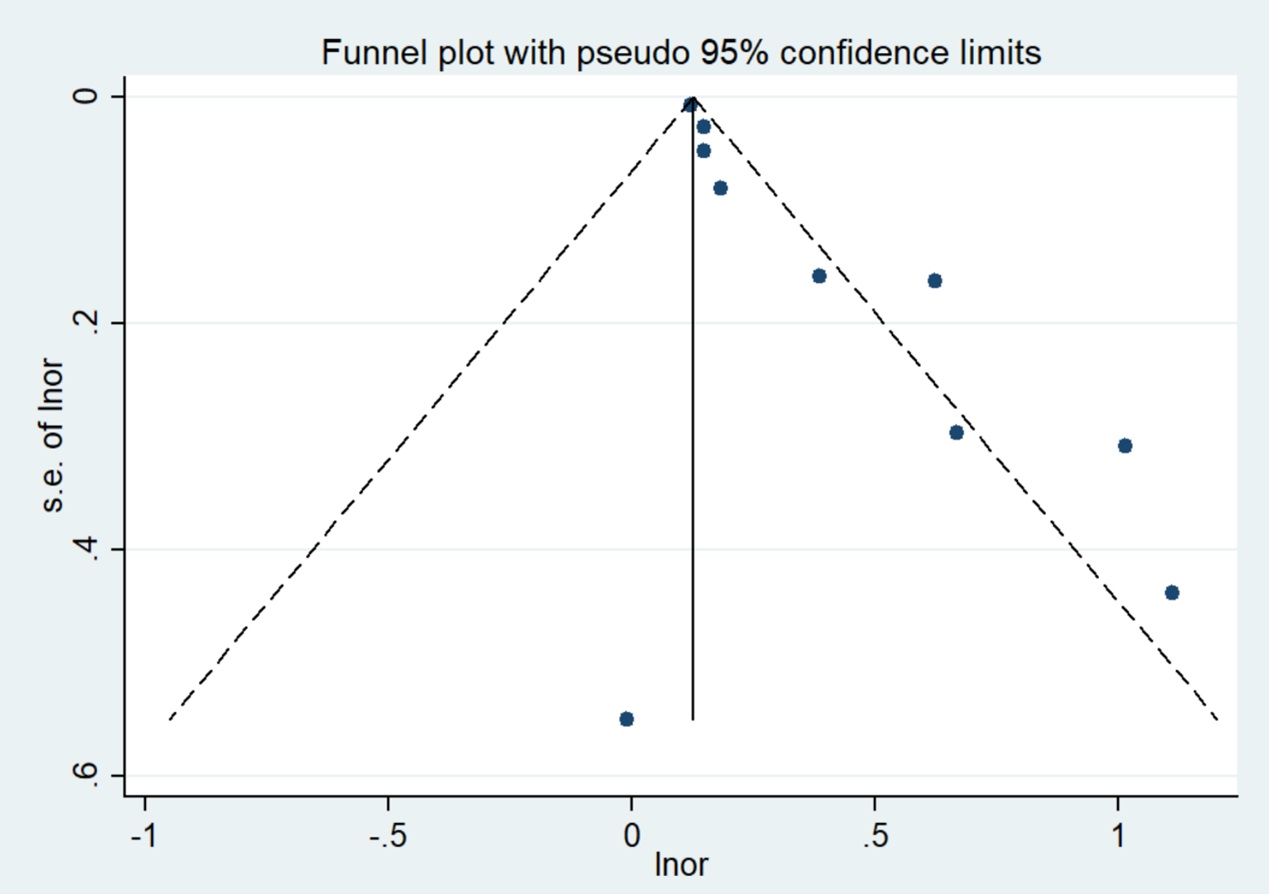

Supplement: Supplementary file 3 [file Image2.JPEG]
