# Supplementary material for: Analysis of the correlation between non-alcoholic fatty liver disease and the risk of colorectal neoplasms
Source: Front Pharmacol. 2022 Nov 9;13:1068432. doi: 10.3389/fphar.2022.1068432 (PMC9682006; doi:10.3389/fphar.2022.1068432)
Supplement: Supplementary file 5 [file DataSheet2.docx]

1. Pubmed

| Search | Query | Items Found |
| --- | --- | --- |
| #1 | "Non-alcoholic Fatty Liver Disease"[Mesh] | 19,113 |
| #2 | (((((Non alcoholic Fatty Liver Disease*[Title/Abstract]) OR (NAFLD[Title/Abstract])) OR (Fatty Liver Nonalcoholic*[Title/Abstract])) OR (Liver Nonalcoholic Fatty*[Title/Abstract])) OR (Nonalcoholic Fatty Liver*[Title/Abstract])) OR (Nonalcoholic Steatohepatiti*[Title/Abstract]) | 30,850 |
| #3 | #1 OR #2 | 33,369 |
| #4 | "Colorectal Neoplasms"[Mesh] | 225189 |
| #5 | (((Colorectal Neoplasm*[Title/Abstract]) OR (Colorectal Tumor*[Title/Abstract])) OR (Colorectal Cancer*[Title/Abstract])) OR (Colorectal Carcinoma*[Title/Abstract]) | 137430 |
| #6 | #4 OR #5 | 262826 |
| #7 | #3 AND #6 | 181 |

1. Cochrane Library

| Search | Query | Items Found |
| --- | --- | --- |
| #1 | MeSH descriptor: [Non-alcoholic Fatty Liver Disease] explode all trees | 1253 |
| #2 | ("Non alcoholic Fatty Liver Disease*"):ti,ab,kw OR (NAFLD):ti,ab,kw OR ("Fatty Liver Nonalcoholic*"):ti,ab,kw OR ("Liver Nonalcoholic Fatty*"):ti,ab,kw OR ("Nonalcoholic Fatty Liver*"):ti,ab,kw (Word variations have been searched) | 3434 |
| #3 | ("Nonalcoholic Steatohepatiti*"):ti,ab,kw (Word variations have been searched) | 938 |
| #4 | #1 OR #2 OR #3 | 3734 |
| #5 | MeSH descriptor: [Colorectal Neoplasms] explode all trees | 9124 |
| #6 | ("Colorectal Neoplasm*"):ti,ab,kw OR ("Colorectal Tumor*"):ti,ab,kw OR ("Colorectal Cancer*"):ti,ab,kw OR ("Colorectal Carcinoma*"):ti,ab,kw (Word variations have been searched) | 16180 |
| #7 | #5 OR #6 | 18807 |
| #8 | #4 AND #7 | 3 |

1. Embase

| Search | Query | Items Found |
| --- | --- | --- |
| #1 | 'nonalcoholic fatty liver'/exp | 57,095 |
| #2 | 'non alcoholic fatty liver disease*':ti,ab,kw OR nafld:ti,ab,kw OR 'fatty liver nonalcoholic*':ti,ab,kw OR 'liver nonalcoholic fatty*':ti,ab,kw OR 'nonalcoholic fatty liver*':ti,ab,kw OR 'nonalcoholic steatohepatiti*':ti,ab,kw | 50,835 |
| #3 | #1 OR #2 | 64,968 |
| #4 | 'colorectal tumor'/exp | 35,074 |
| #5 | 'colorectal neoplasm*':ti,ab,kw OR 'colorectal tumor*':ti,ab,kw OR 'colorectal cancer*':ti,ab,kw OR 'colorectal carcinoma*':ti,ab,kw | 205,305 |
| #6 | #4 OR #5 | 214,671 |
| #7 | #3 AND #6 | 379 |
