# Supplementary material for: Analysis of the correlation between non-alcoholic fatty liver disease and the risk of colorectal neoplasms
Source: Front Pharmacol. 2022 Nov 9;13:1068432. doi: 10.3389/fphar.2022.1068432 (PMC9682006; doi:10.3389/fphar.2022.1068432)
Supplement: Supplementary file 6 [file DataSheet1.docx]

**Newcastle-Ottawa Quality Assessment Scale for cohort studies included in this meta-analysis.**

**References Selection Comparability Outcome Overall quality score**

Yoon Jin Choi et al. (2020) 🟊🟊🟊🟊 🟊 🟊🟊🟊 8

Gi-Ae Kim et al. (2020) 🟊🟊🟊🟊 🟊🟊 🟊🟊🟊 9

Zhengyu Wang et al. (2020) 🟊🟊🟊🟊 🟊🟊 🟊🟊🟊 9

Young In Lee et al. (2011) 🟊🟊🟊🟊 🟊🟊 🟊🟊🟊 9

Vincent Wai-Sun Wong et al. (2011) 🟊🟊🟊🟊 🟊🟊 🟊🟊🟊 9

Xian-Feng Lin et al. (2014) 🟊🟊🟊🟊 🟊🟊 🟊🟊🟊 9

Hokyou Lee et al. (2022) 🟊🟊🟊🟊 🟊🟊 🟊🟊🟊 9

Yuri Cho et al. (2019) 🟊🟊🟊🟊 🟊🟊 🟊🟊🟊 9

Jung-Min Lee et al. (2020) 🟊🟊🟊🟊 🟊 🟊🟊🟊 8

A. Stadlmayr et al. (2011) 🟊🟊🟊🟊 🟊 🟊🟊🟊 8

Masahide Hamaguchi et al. (2019) 🟊🟊🟊🟊 🟊 🟊🟊🟊 8

Nam Hee Kim et al. (2021) 🟊🟊🟊 🟊 🟊🟊🟊 7

Birju D. Bhatt et al. (2015) 🟊🟊🟊 🟊 🟊🟊🟊 7

Eun Young Ze et.al. (2018) 🟊🟊🟊 🟊🟊 🟊🟊🟊 8
